# The state of health in Belgium, 1990–2019: a benchmarking analysis based on the Global Burden of Disease 2019 study

**DOI:** 10.1186/s13690-022-00976-2

**Published:** 2022-10-18

**Authors:** Jinane Ghattas, Vanessa Gorasso, Robby De Pauw, Sophie Thunus, Niko Speybroeck, Brecht Devleesschauwer

**Affiliations:** 1 Institute of Health and Society (IRSS), Université Catholique de Louvain, Brussels, Belgium; 2 Lifestyle and Chronic Diseases, Department of Epidemiology and Public Health, Rue Juliette Wytsmanstraat 14, 1050 Sciensano Brussels, Belgium; 3 Department of Public Health and Primary Care, Ghent University, Ghent, Belgium; 4 Department of Rehabilitation Sciences, Faculty of Medicine and Health Sciences, Ghent University, Ghent, Belgium; 5 Department of Translational Physiology, Infectiology and Public Health, Ghent University, Merelbeke, Belgium

**Keywords:** Burden of disease, Disability-Adjusted Life Years, Public health monitoring

## Abstract

**Background:**

In a context of decreasing resources and growing health needs, evidence-based health and care policies are essential. This study aims to assess the health trends in Belgium between 1990 and 2019, to compare the Belgian health status to that of the EU-15 countries, and to identify the main drivers in trends over time and country differences within the EU-15.

**Methods:**

We extracted estimates from the GBD 2019 study via the GBD results tool and visualization tools. We compared the Belgian health status with 14 European Union comparator countries between 1990 and 2019, and decomposed the time trends and country differences into the unique contributions of the different underlying causes of death and disability.

**Results:**

Life expectancy (LE) in Belgium improved significantly between 1990 and 2019 for both men and women. Belgium age-standardised mortality rates dropped significantly for men (-40%) and women (-33%) between 1990 and 2019. Overall, Belgium age-standardised disability-adjusted life year (DALY) rates dropped by 23%. This decrease is mainly due to decreasing trends in age-standardised years of life lost (YLL) rates while age-standardised years lived with disability (YLD) rates remained stable. Compared to EU-15, Belgium’s ranking in terms of age-standardised DALY rates worsened for both men and women in 2019. Self-harm and falls are major causes of disease burden, with DALY rates that are higher than in many other EU-15 countries, indicating a realistic potential for improvement. Lung cancer DALY rates remain worrisome for men, and even show an increasing trend for women. Increasing trends of headache disorders, drug use disorders, and diabetes, require further attention.

**Conclusion:**

Non-communicable diseases remain the main contributors for health burden in Belgium, with disability accounting for an increasingly larger share of the disease burden. Despite considerable improvements, Belgium’s ranking for DALYs decreased between 1990 and 2019 compared to the EU-15. This study identified priority causes of disease burden based on their contributions to current evolutions and EU-15 differences. Since many of these causes are considered to be avoidable, primary and secondary prevention are crucial elements for reducing the burden of disease on the healthcare system.

## Background

As in many high-income countries, ageing of the population is putting additional pressure on the Belgian healthcare system [1]. Currently, 16% of the population is above 65 years, while this percentage is projected to increase to 18% by 2040 [2]. Older age is often accompanied by increasing morbidity and frailty, which require appropriate care and health infrastructures [3]. According to the most recent Health Interview Survey, 29% of all adults live with a chronic disease; among the population aged 75 years and over, this prevalence reaches 44%, and is typically characterised by a state of multimorbidity [4]. In Belgium, the proportion of daily smokers is in continuous decrease since 1997, and is below the EU-15 average. While smoking decreased, alcohol consumption and obesity remained important public health risks [5]. Over the last two decades, Belgium has implemented many reforms to address the needs of an aging population and increasing chronic disease burden. These initiatives and reforms cover healthcare organisation such as the mental healthcare reforms [6], prevention such as national policies for promoting healthy eating habits, taxation on sugar-sweetened beverages [7, 8], and improving financial accessibility and efficiency [9]. Despite these efforts, many international reports still highlight a poor state of health of Belgium compared to its peers [10].

In a context of decreasing resources and growing health needs of the population, evidence-based health and care policies are essential. This involves independent and objective assessments of the population’s health state to be able to set priorities, with consistent and comparable data on mortality and morbidity [11]. The Global Burden of Disease (GBD) study offers a comprehensive framework for decision-makers (at the local, regional, national, and global level) by estimating trends in, and drivers of, population health. This allows decision-making processes to be based on internally consistent evidence, obtained via a systematic quantification of the comparative magnitude of health loss from diseases, injuries, and risks by age, sex, and population over time. Today, the GBD study covers 204 countries and territories and includes a vast number of parameters, i.e. 369 diseases and injuries, 3,473 sequelae of these diseases and injuries, and 87 risks or combinations of risks using 281,586 data sources [12–14].

Using the results of Global Burden of Disease 2019, this study aims to assess the health trends in Belgium between 1990 and 2019, to compare the Belgian health status to that of the EU-15 countries, and to identify the main drivers in trends over time and country differences within the EU-15.

## Methods

We extracted estimates from the GBD 2019 study via the GBD results tool [15] and visualization tool [16]. We focused on life expectancy (LE), mortality rates, years of life lost (YLLs), years lived with disability (YLDs) and disability-adjusted life years (DALYs) in Belgium between 1990 and 2019 for level 3 causes and risk factors [12].

The GBD generates data on the basis of a comparative descriptive approach of health status in the world according to age, sex and geographical locations on different health metrics. The latest published version is the GBD 2019 that looks at 369 diseases and injuries and 84 risk factors in 204 countries. GBD 2019 follows the Guidelines for Accurate and Transparent Health Estimates Reporting [17].

YLLs measure premature death caused by a specific disease or injury. It is the product of the number of deaths multiplied by the residual standard life expectancy at death [12]. YLDs are calculated by multiplying the prevalence of diseases or injuries by their (severity-weighted) disability weight [12]. DALYs are the sum of YLLs and YLDs, accounting for the years of healthy life lost due to premature death and disability [18]. We extracted age-standardised rates (per 100,000), based on GBD’s global population standard, for comparing estimates between genders, time periods and countries. To assess the significance of differences in rates, we assessed whether or not the 95% uncertainty intervals (UIs) overlapped: non-overlapping UIs were considered indicative of significant differences, whereas overlapping UIs were considered inconclusive. Age-standardisation is a statistical method whereby rates are adjusted according to the population weight of each age group. GBD provides a set of standard population weights to be used for age-standardisation [12, 13]. This statistical technique is particularly relevant in countries with aging population such as Belgium.

### Benchmarking

We compared the Belgian health status with 14 European Union comparator countries: Austria, Denmark, Finland, France, Germany, Greece, Ireland, Italy, Luxembourg, Netherlands, Portugal, Spain, United Kingdom and Sweden. The selected countries have similar economic, demographic and social conditions as Belgium, and are jointly referred to as the EU-15 countries. We compared Belgium to the average of the EU-15 countries in terms of LE, mortality, YLL, YLD, and DALY rates. Furthermore, we decomposed the differences in mortality, YLL, YLD, and DALY rates between Belgium and each of the other EU-15 countries, allowing to identify the main causes of mortality and disability for which Belgium performs inferior or superior, across the set of comparator countries. Specifically, we counted, for each cause, the number of countries that perform better than Belgium, and the number of countries that perform worse than Belgium. We then report the cause with the highest number of better-performing countries, and the cause with the highest number of worse-performing countries.

### Decomposition of time trends and country differences

In addition to reporting the GBD estimates for Belgium, we decomposed the time trends and country differences into the unique contributions of the different underlying causes of death and disability. For any given country, year and sex, the overall (age-standardised) mortality, YLD, YLL, or DALY rate is the sum of the cause-specific rates. Mathematically, it then follows that a difference in overall rates (e.g., between two time periods or between two countries), corresponds to the sum of differences in cause-specific rates. This decomposition allows us to pinpoint the main drivers of time trends or differences among country.

## Results

### Life expectancy

**Fig. 1:**
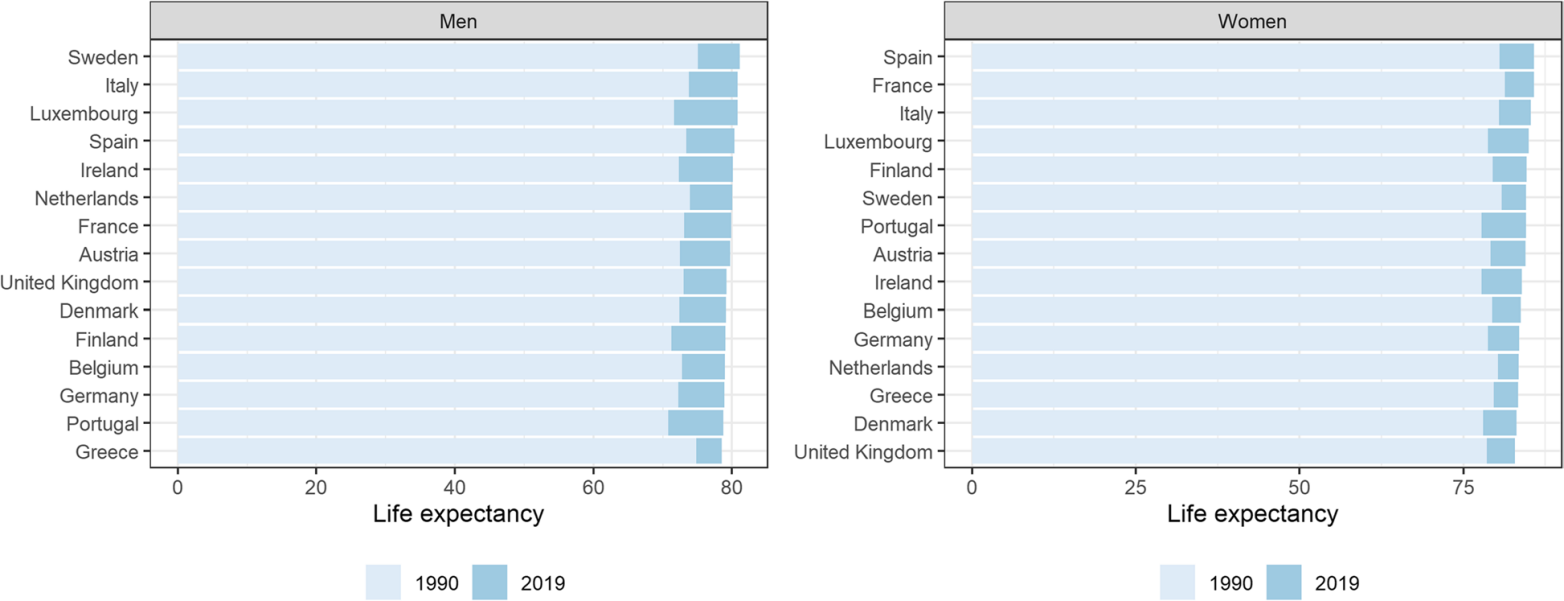
Life expectancy (LE) at birth in the EU-15, in 1990 and 2019, for men and women

In 2019, LE in Belgium was 79 years [95% UI 78.7–79.2] for men and 84 years [95% UI 83.6–84] for women. Between 1990 and 2019, LE at birth increased significantly for both men (+ 6.2 years) and women (+ 4.3 years). In 2019, life expectancy in Belgium was slightly below the EU-15 average for both men (-0.7) and women (-0.5) (EU-15 LE in men: 79.7 years [95% UI 79.4–80]; EU-15 LE in women 84.3 years [95% UI 84–84.5] – these differences were significant. Across the EU-15 countries, LE in Belgian men and women ranked 12^th^ and 10^th^, respectively (Fig. 1). This is worse compared to the LE among men and women in 1990, where Belgium ranked 8^th^ for LE in men and women.

### Mortality rates

In men, age-standardised mortality rates for the top five causes of death observed in 2019 were ischaemic heart disease (74 deaths [95% UI: 68–79] per 100,000), tracheal, bronchus, and lung cancer (henceforth referred to as “lung cancer”) (55 deaths [95% UI: 51–58] per 100,000), chronic obstructive pulmonary disease (COPD) (37 deaths [95% UI: 32–42] per 100,000), stroke (35 deaths [95% UI: 31–39] per 100,000), and lower respiratory infections (27 deaths [95% UI: 24–30] per 100,000). The top five causes of mortality identified in women were ischaemic heart disease (40 deaths [95% UI: 34–44] per 100,000), stroke (30 deaths [95% UI: 26–34] per 100,000), breast cancer (23 deaths [95% UI: 21–25] per 100,000), Alzheimer’s disease and other dementias (henceforth referred to as “dementia” (22 deaths [95% UI: 6–56] per 100,000), and lung cancer (19 deaths [95% UI: 18–21] per 100,000).

#### Evolution 1990–2019

**Fig. 2:**
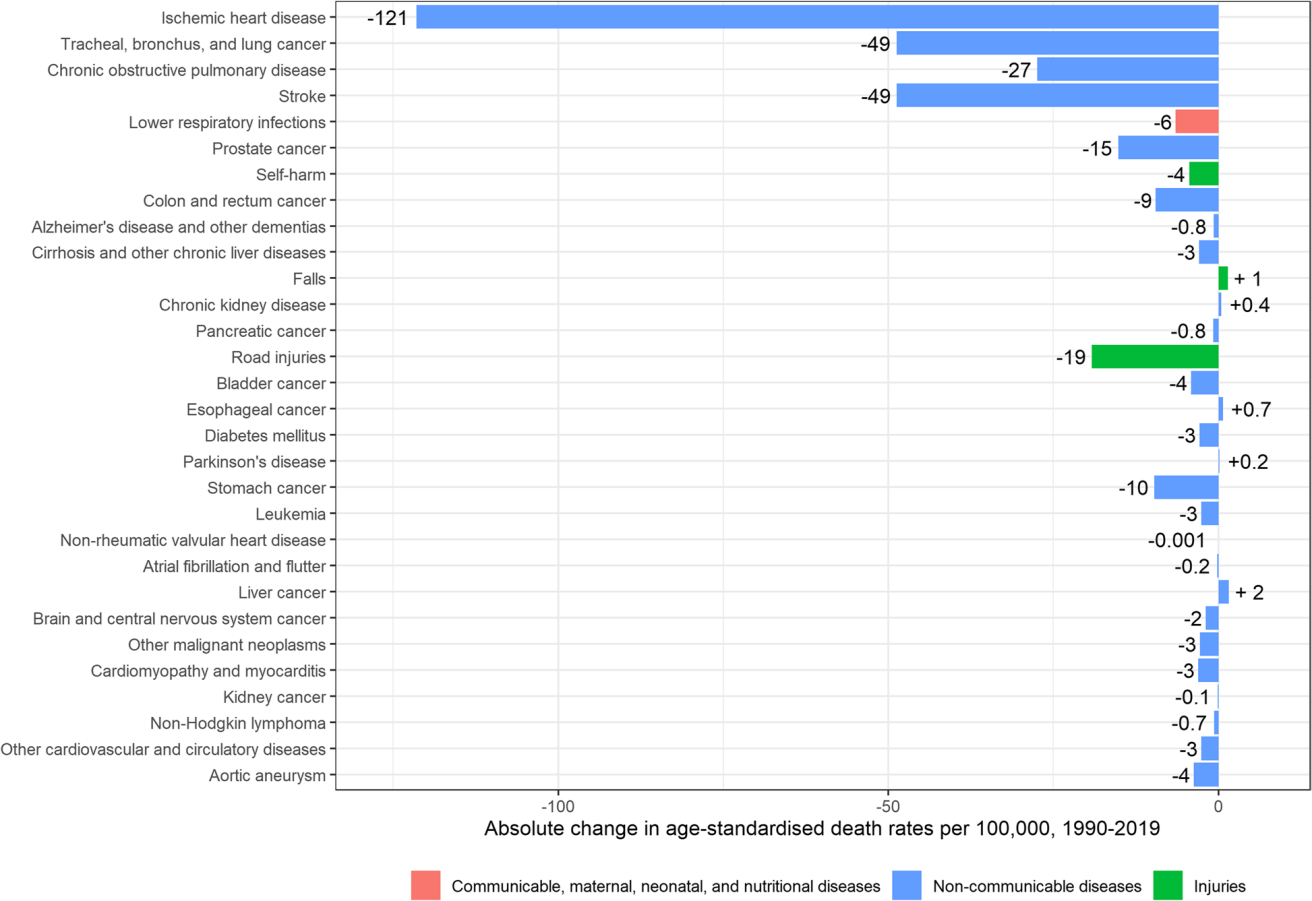
Absolute change in age-standardised death rates per 100,000 in men, 1990–2019, Belgium

Age-standardised mortality rates for men significantly dropped from 924 [95% UI: 916–931] per 100,000 in 1990 to 559 [95%UI: 546–572] per 100,000 in 2019 (-365 deaths per 100,000; -40%). The main contributors to this significant decrease were ischaemic heart disease (-121 deaths per 100,000), lung cancer (-49 deaths per 100,000), stroke (-49 deaths per 100,000), COPD (-27 deaths per 100,000), and road injuries (-19 deaths per 100,000) (Fig. 2).

**Fig. 3:**
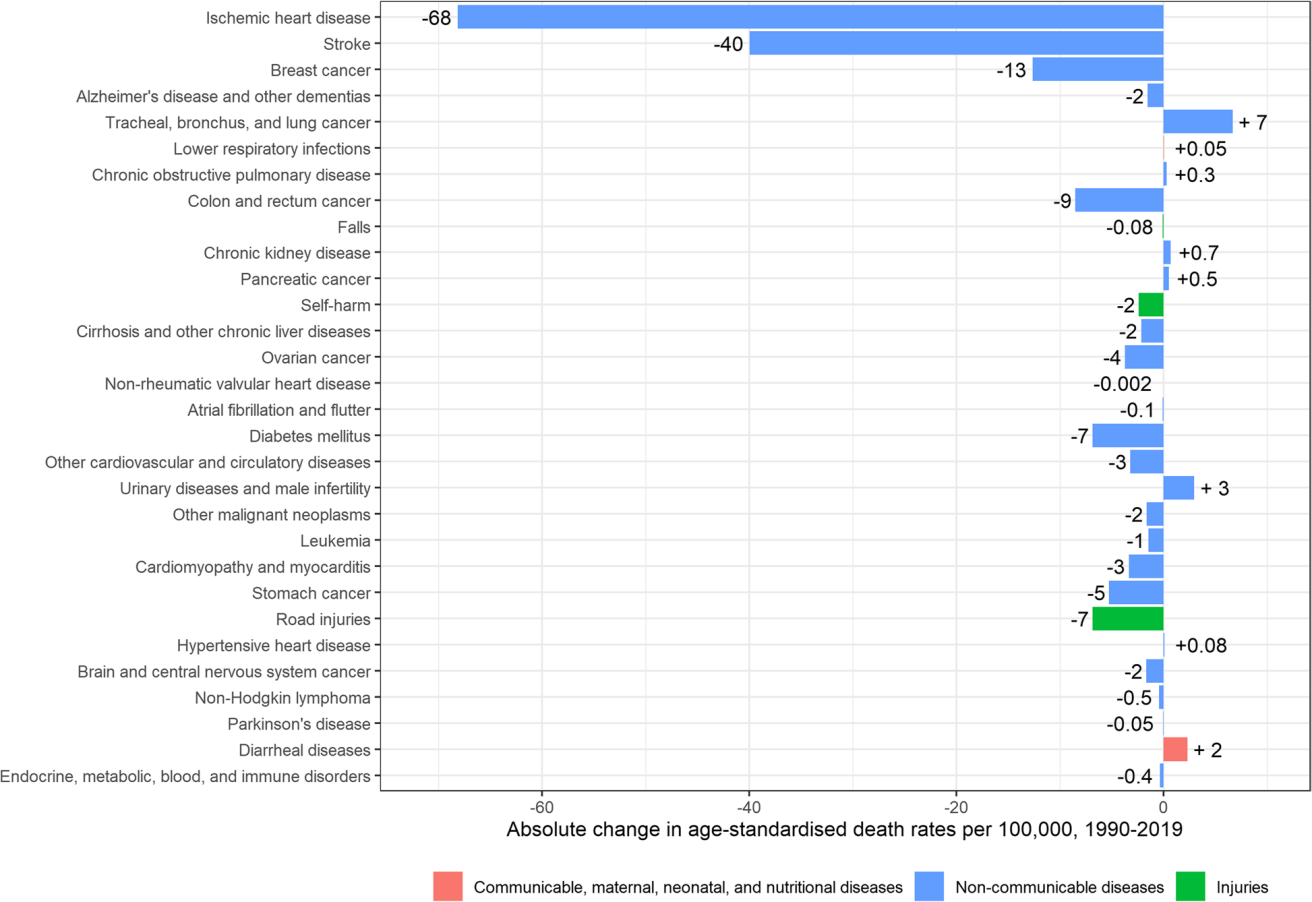
Absolute change in age-standardised death rates per 100,000 in women, 1990–2019, Belgium

Age-standardised mortality rates for women also decreased significantly, from 541 [95% UI: 536–546] per 100,000 in 1990 to 362 [95% UI: 354–371] per 100,000 in 2019 (-179 deaths per 100,000; -33%). The main contributors to this significant decrease were ischaemic heart disease (-68 deaths per 100,000), stroke (-40 deaths per 100,000), breast cancer (-13 deaths per 100,000), colorectal cancer (-9 deaths per 100,000), and diabetes mellitus (-7 deaths per 100,000). Deaths caused by lung cancer however significantly increased from 13 deaths [95% UI: 12–14] per 100,000 to 20 deaths [95% UI: 18–21] per 100,000 (+ 7 deaths per 100,000) (Fig. 3).

#### Benchmarking

In 2019, the age-standardised mortality rate was higher than the EU-15 average for both Belgian men (BE: 559 deaths [95% UI: 546–572] – EU-15: 526 deaths [95% UI: 513–541]), and women (BE: 362 deaths [95% UI: 354–371] – EU-15: 348 deaths [95% UI: 340–357]). Across the EU-15 countries, Belgium ranked 12^th^ and 10^th^ in terms of age-standardised mortality rates for men and women, respectively. This is a worsening compared to 1990, when Belgium ranked 9^th^ and 6^th^, respectively.

In Belgium, men performed significantly worse compared to the EU-15 average in terms of lung cancer (+ 13 deaths per 100,000), lower respiratory infections (+ 9 deaths per 100,000), COPD (+ 8 deaths per 100,000), self-harm (+ 8 deaths per 100,000), and falls (+ 3 deaths per 100,000). Men however performed significantly better in terms of ischaemic heart disease (-12 deaths per 100,000) and hypertensive heart disease (-4 deaths per 100,000). Belgian women performed significantly worse in terms of lower respiratory infections (+ 5 deaths per 100,000), self-harm (+ 4 deaths per 100,000) and breast cancer (+ 3 deaths per 100,000) but significantly better in terms of ischaemic heart disease (-8 deaths per 100,000) and hypertensive heart disease (-4 deaths per 100,000).

Compared to each individual EU-15 country, Belgian men performed worse than men in 8 other countries in terms of lung cancer, and better than men in 7 countries in terms of ischaemic heart disease. Belgian women performed worst in terms of lower respiratory infections (6 countries), and best in terms of ischaemic heart disease (5 countries).

### Years of life lost

In men, in 2019, age-standardised YLL rates were primarily due to ischaemic heart disease (1,221 YLLs [95% UI: 1,151–1,285] per 100,000), lung cancer (1,141 YLLs [95% UI: 1,071–1,208] per 100,000), self-harm (951 YLLs [95% UI: 895–1,014] per 100,000), COPD (543 YLLs [95% UI: 479–623] per 100,000), and stroke (519 YLLs [95% UI: 471–564] per 100,000). The major causes of age-standardised YLLs in women were breast cancer (557 YLLs [95% UI: 520–599] per 100,000), ischaemic heart disease (529 YLLs [95% UI: 472–573] per 100,000), lung cancer (471 YLLs [95% UI: 435–510] per 100,000), stroke (416 YLLs [95% UI: 367–457] per 100,000) and self-harm (336 YLLs [95% UI: 313–360] per 100,000).

#### Evolution 1990–2019

**Fig. 4:**
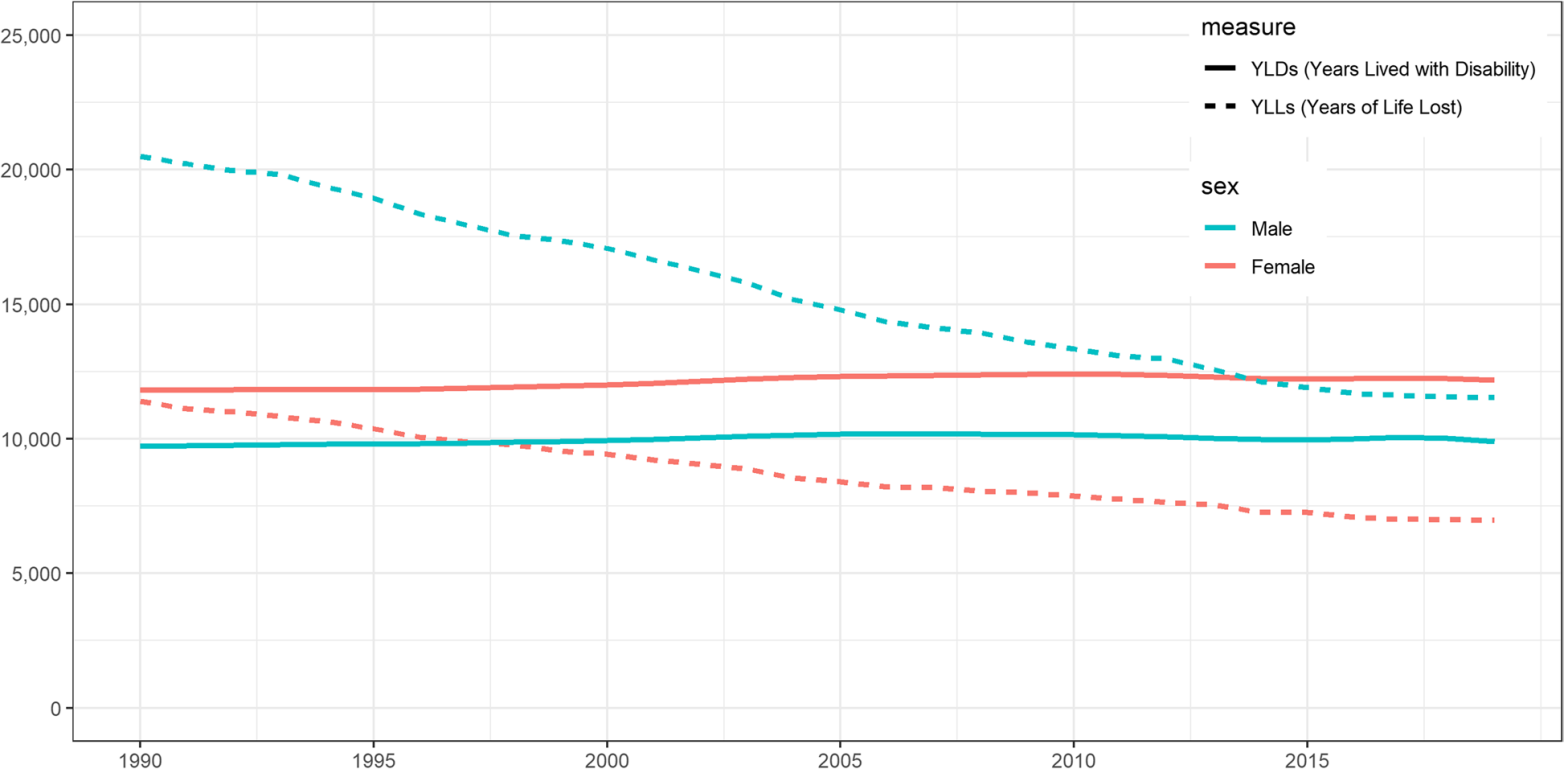
Evolution of age-standardised Years Lived with Disability (YLDs) and Years of Life Lost (YLLs) rates per 100,000 in men and women, Belgium, 1990–2019

**Fig. 5:**
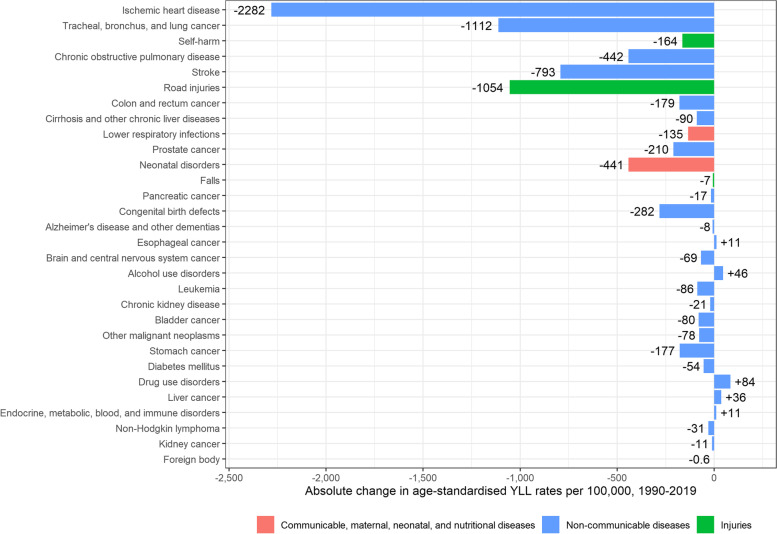
Absolute change in age-standardised Years of Life Lost (YLLs) rates per 100,000 in men, 1990–2019, Belgium

Age-standardised YLL rates for men significantly decreased from 20,493 [95% UI: 20,296–20,692] per 100,000 in 1990, to 11,526 [95%UI: 11,161–11,930] per 100,000 in 2019 (-8967 YLLs per 100,000; -44%) (Fig. 4). The main contributors to the significant YLL decrease in men were ischaemic heart disease (-2,283 YLLs per 100,000), lung cancer (-1,112 YLLs per 100,000), road injuries (-1,054 YLLs per 100,000), stroke (-793 YLLs per 100,000), and COPD (-442 YLLs per 100,000). YLLs however increased between 1990 and 2019 for drug use disorders (+ 84 YLLs per 100,000), alcohol use disorders (+ 46 YLLs per 100,000), and liver cancer (+ 36 YLLs per 100,000) (Fig. 5). Drug use disorders are related to the use of opioids, amphetamines, cocaine, cannabis and others [19].

**Fig. 6:**
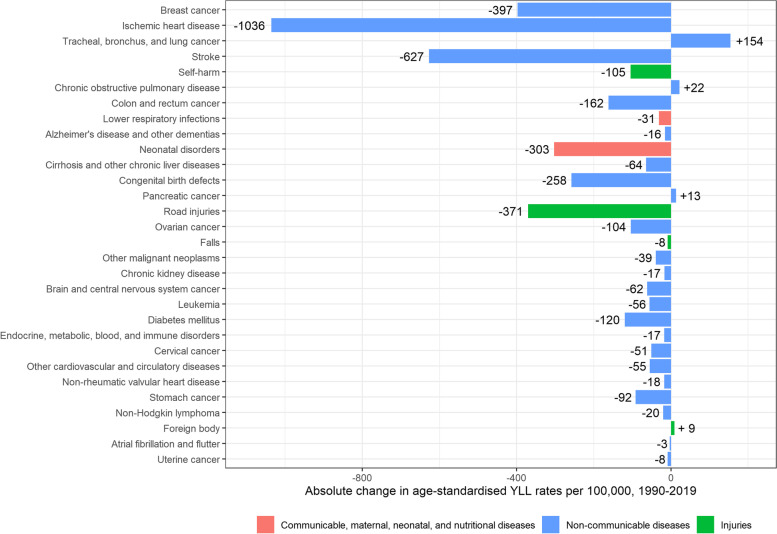
Absolute change in age-standardised Years of Life Lost (YLLs) rates per 100,000 in women, 1990–2019, Belgium

Age-standardised YLL rates for women also decreased significantly, from 11,396 [95% UI: 11,271–11,524] per 100,000 in 1990, to 6,962 [95%UI: 6,740–7,208] per 100,000 in 2019 (-4,434 deaths per 100,000; -40%) (Fig. 4). The main causes of the significant decrease in YLLs in women were ischaemic heart disease (-1,036 YLLs per 100,000), stroke (-627 YLLs per 100,000), breast cancer (-397 YLLs per 100,000), road injuries (-371 YLLs per 100,000) and neonatal disorders (-303 YLLs per 100,000). YLLs caused by lung cancer, however, significantly increased from 317 YLLs [95% UI: 301–333] per 100,000 to 471 YLLs [95% UI: 435–510] per 100,000 (+ 154 per 100,000; + 54%) (Fig. 6).

#### Benchmarking

In 2019, the age-standardised YLL rate in Belgium was higher than the EU-15 average for both men (BE: 11,526 YLLS [95%UI: 11,161–11,930] – EU-15: 10,737 YLLs [95% UI: 10,368–11,153]), and women (BE: 6,962 YLLs [95%UI: 6,740–7,208] – EU-15: 6,497 YLLs [95% UI: 6274–6750]). Across the EU-15 countries, Belgium ranked 12^th^ and 10^th^ in terms of age-standardised YLL rates for men and women, respectively. This is a worsening compared to 1990, where Belgium ranked 8^th^ for both men and women.

Belgian men performed significantly worse compared to the EU-15 average in terms of self-harm (+ 358 YLLs per 100,000), lung cancer (+ 259 YLLs per 100,000), COPD (+ 148 YLLs per 100,000), road injuries (+ 113 YLLs per 100,000), and lower respiratory infections (+ 107 YLLs per 100,000). Men however performed significantly better in terms of ischaemic heart disease (-196 YLLs per 100,000) and drug use disorders (-56 YLLS per 100,000). Belgian women performed significantly worse in terms of self-harm (+ 150 YLLs per 100,000), and breast cancer (+ 76 YLLs per 100,000) but significantly better in terms of ischaemic heart disease (-63 YLLs per 100,000) and hypertensive heart disease (-40 YLLs per 100,000).

Compared to the individual EU-15 countries, in terms of self-harm, Belgian men and women performed worse than 9 and 8 countries respectively, and better than 6 countries in terms of ischaemic heart disease for both men and women.

### Years lived with disability (YLD)

In 2019, the main causes of age-standardised YLD rates in men were low back pain (898 YLDs [95% UI: 624–1,215] per 100,000), falls (596 YLDs [95% UI: 408–855] per 100,000), headache disorders (576 YLDs [95% UI: 87–1,309] per 100,000), depressive disorders (478 YLDs [95% UI: 329–660] per 100,000), and diabetes mellitus (424 YLDs [95% UI: 277–606] per 100,000). The main causes of age-standardised YLD rates in women were headache disorders (1,206 YLDs [95% UI: 184–2,802] per 100,000), low back pain (1,184 YLDs [95% UI: 833–1,595] per 100,000), gynaecological disorders (1,129 YLDs [95% UI: 764–1,568] per 100,000), depressive disorders (762 YLDs [95% UI: 517–1,069] per 100,000), and falls (616 YLDs [95% UI: 427–862] per 100,000).

#### Evolution 1990–2019

**Fig. 7:**
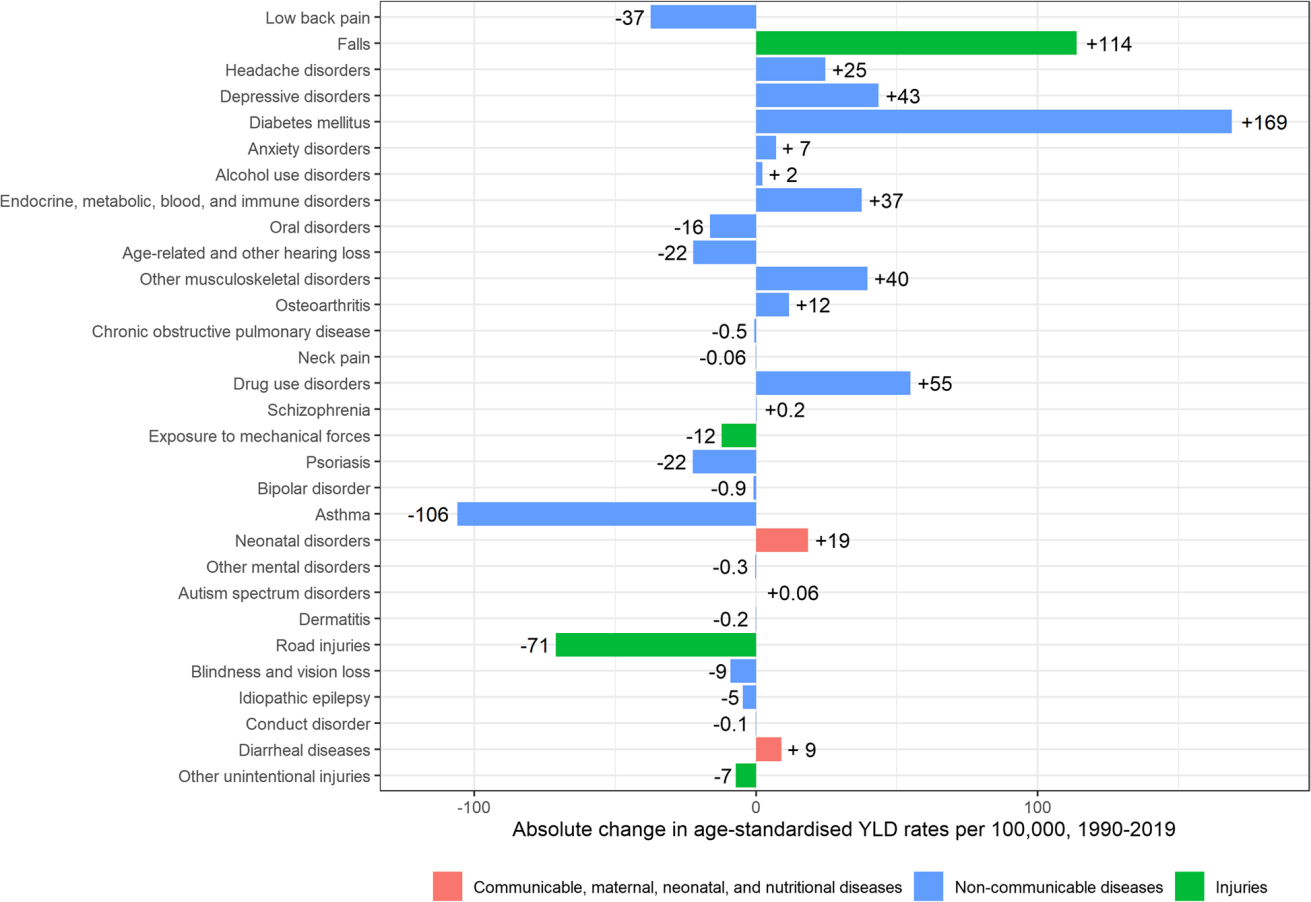
Absolute change in age-standardised Years Lived with Disability (YLDs) rates per 100,000 in men, 1990–2019, Belgium

Age-standardised YLD rates for men increased, albeit with overlapping UIs, from 9,726 [95% UI: 7,192–12,669] per 100,000 in 1990, to 9,901 [95%UI: 7,332- 12,889] per 100,000 in 2019 (+ 175 YLDs per 100,000; + 1.8%) (Fig. 4). The main contributors to the increase in YLDs between 1990 and 2019 in men were diabetes mellitus (+ 169 YLDs per 100,000), falls (+ 114 YLDs per 100,000), drug use disorders (+ 55 YLDs per 100,000), depressive disorders (+ 43 YLDs per 100,000), and other musculoskeletal disorders (+ 40 YLDs per 100,000). In the same period, YLDs caused by asthma and road injuries have decreased (-106 YLDs per 100,000 and -71 YLDs per 100,000, respectively) (Fig. 7).

**Fig. 8:**
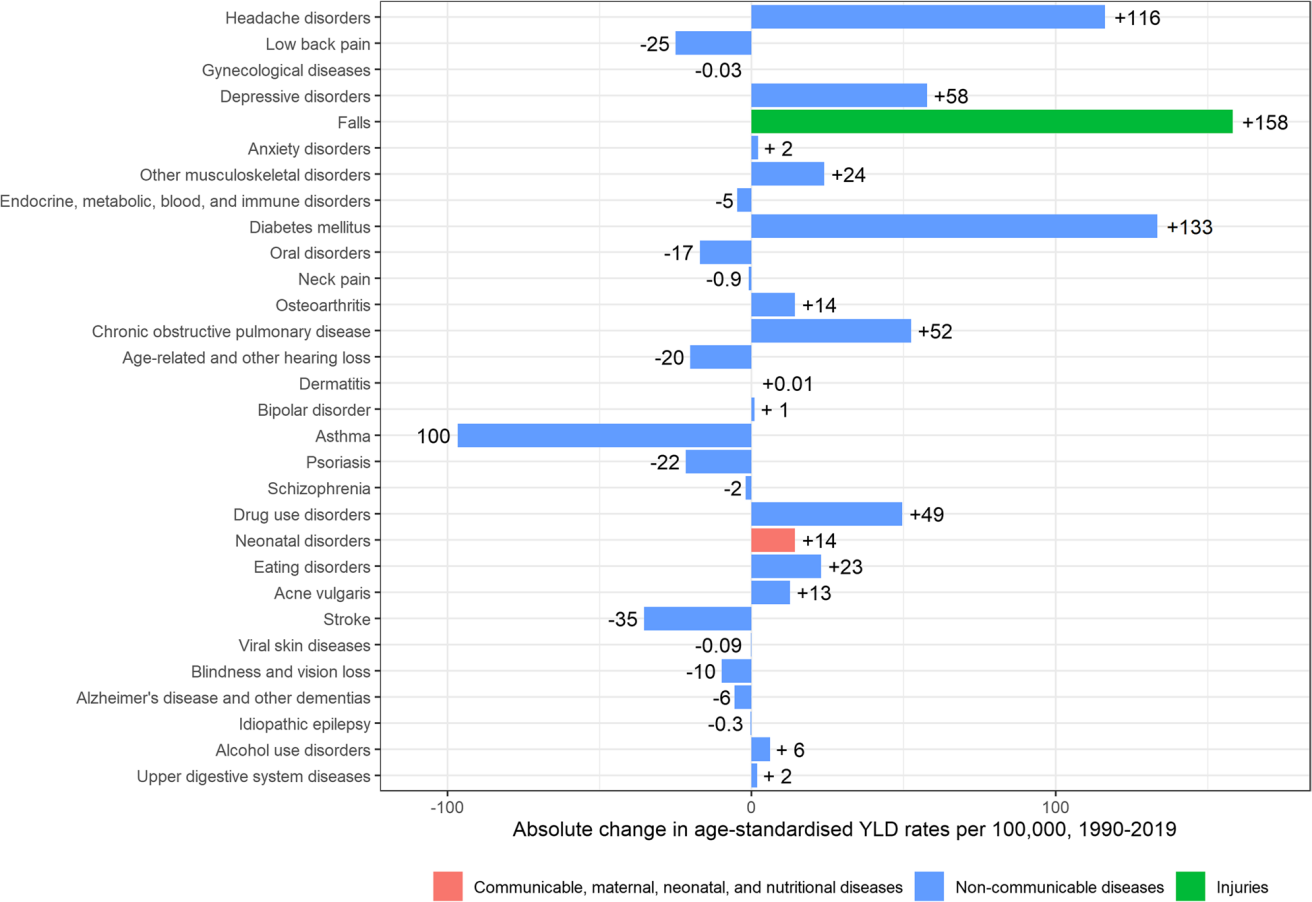
Absolute change in age-standardised Years Lived with Disability (YLDs) rates per 100,000 in women, 1990–2019, Belgium

As for men, there was an increase in age-standardised YLD rates for women, from 11,808 [95% UI: 8,623–15,448] per 100,000 in 1990, to 12,178 [95%UI: 8,886–15,797] per 100,000 in 2019 (+ 371 YLDs per 100,000; + 3.1%) (Fig. 4). The main contributors to the increase in YLDs in women were falls (+ 158 YLDs per 100,000), diabetes mellitus (+ 133 YLDs per 100,000), headache disorders (+ 116 YLDs per 100,000), depressive disorders (+ 58 YLDs per 100,000), and COPD (+ 52 YLDs per 100,000). In the same period, YLDs caused by asthma (-97 YLDs per 100,000) decreased (Fig. 8).

#### Benchmarking

In 2019, the age-standardised YLD rate in Belgium was higher, albeit with overlapping UIs, than the EU-15 average for both men (BE: 9,900 YLDs [95%UI: 7,332- 12,889] – EU-15: 9,571 YLDs [95% UI: 7,082–12,403]), and women (BE: 12,178 YLDs [95%UI: 8,886–15,797] – EU-15: 12,023 YLDs [95% UI: 8,821–15,684]). Across the EU-15 countries, Belgium ranked 14^th^ and 12^th^ in terms of age-standardised YLD rates for men and women, respectively. This is a worsening compared to 1990, where Belgium ranked 9^th^ and 5^th^ for men and women, respectively.

Belgian men performed worse compared to the EU-15 average in terms of endocrine, metabolic, blood, and immune disorders (+ 119 YLDs per 100,000), falls (+ 110 YLDs per 100,000), headache disorders (+ 83 YLDs per 100,000), osteoarthritis (+ 52 YLDs per 100,000) and, COPD (+ 49 YLDs per 100,000). Men performed better, albeit with overlapping UIs, in terms of asthma (-51 YLDs per 100,000) and neck pain (-50 YLDs per 100,000). Belgian women performed worse in terms of headache disorders (+ 225 YLDs per 100,000), falls (+ 174 YLDs per 100,000), gynaecological disorders (+ 138 YLDs per 100,000), oral disorders (+ 50 YLDs per 100,000), and COPD (+ 37 YLDs per 100,000). Women performed better, albeit with overlapping UIs, in terms of anxiety disorders (-122 YLDs per 100,000) and depressive disorders (-105 YLDs per 100,000).

Compared to the individual EU-15 countries, Belgian men performed worst in terms of falls (6 countries), and best in terms of low back pain (3 countries). Belgian women performed worst in terms of headache disorders (8 countries), and best in terms of anxiety disorders (7 countries).

### Disability-adjusted life years (DALY)

In 2019, the main causes of age-standardised DALY rates in men were ischaemic heart disease (1,291 DALYs [95% UI: 1,221–1,362] per 100,000), lung cancer (1,156 DALYs [95% UI: 1,084–1,223] per 100,000), self-harm (968 DALYs [95% UI: 913–1,034] per 100,000), low back pain (898 DALYs [95% UI: 624–1,215] per 100,000), and falls (840 DALYs [95% UI: 648–1,094] per 100,000) (Table 1). The main causes of age-standardised DALY rates in women were headache disorders (1,206 DALYs [95% UI: 184–2,801] per 100,000), low back pain (1,184 DALYs [95% UI: 833–1,595] per 100,000), gynaecological disorders (1,129 DALYs [95% UI: 765–1,569] per 100,000), depressive disorders (762 DALYs [95% UI: 517–1,069] per 100,000), and falls (751 DALYs [95% UI: 558–998] per 100,000) (Table 2).

**Table 1:** Age-standardised Disability-Adjusted Life Years (DALYs) per 100,000 by cause, men, 1990 and 2019, Belgium

	**Belgium, 1990**				**Belgium, 2019**				
**Causes**	**DALY rank**	**DALY**^**a**^** rate**	**LB 95% UI**	**UB 95% UI**	**DALY rank**	**DALY**^**a**^** rate**	**LB 95% UI**	**UB 95% UI**	**Absolute DALYs changes**
Ischaemic heart disease	1	3599	3470	3715	1	1291	1222	1362	-2307
Tracheal, bronchus, and lung cancer	2	2277	2197	2356	2	1156	1084	1223	-1121
Self-harm	6	1133	1094	1171	3	968	913	1034	-165
Low back pain	7	935	653	1280	4	898	624	1215	-37
Falls	9	733	579	942	5	840	648	1094	107
Chronic obstructive pulmonary disease	5	1240	1148	1321	6	798	714	890	-442
Stroke	4	1439	1362	1512	7	608	553	660	-832
Road injuries	3	1731	1661	1805	8	606	558	653	-1125
Headache disorders	13	551	86	1239	9	576	87	1309	25
Diabetes mellitus	17	441	352	550	10	556	409	743	115

^a^Age-standardised DALY per 100,000; *LB* Lower bound, *UB* Upper bound, *UI* Uncertainty interval

**Table 2:** Age-standardised Disability-Adjusted Life Years (DALYs) per 100,000 by cause, women, 1990 and 2019, Belgium

	**Belgium, 1990**				**Belgium, 2019**				
**Causes**	**DALY rank**	**DALY**^a^** rate**	**LB 95% UI**	**UB 95% UI**	**DALY rank**	**DALY**^a^** rate**	**LB 95% UI**	**UB 95% UI**	**Absolute DALYs changes**
Headache disorders	5	1090	186	2511	1	1206	184	2802	116
Low back pain	2	1209	849	1633	2	1184	833	1595	-25
Gynaecological disorders	4	1129	764	1569	3	1129	765	1569	0
Depressive disorders	7	704	494	953	4	762	517	1069	58
Falls	10	601	457	790	5	751	558	998	150
Breast cancer	6	1029	985	1073	6	630	580	693	-400
Anxiety disorders	11	570	382	810	7	572	374	815	2
Ischaemic heart disease	1	1619	1510	1691	8	571	511	618	-1049
Stroke	3	1196	1109	1263	9	534	481	589	-662
Chronic obstructive pulmonary disease	17	412	360	466	10	487	406	552	75

^a^Age-standardised DALY per 100,000, *LB* Lower bound, *UB* Upper bound, *UI* Uncertainty interval

#### Evolution 1990–2019

**Fig. 9:**
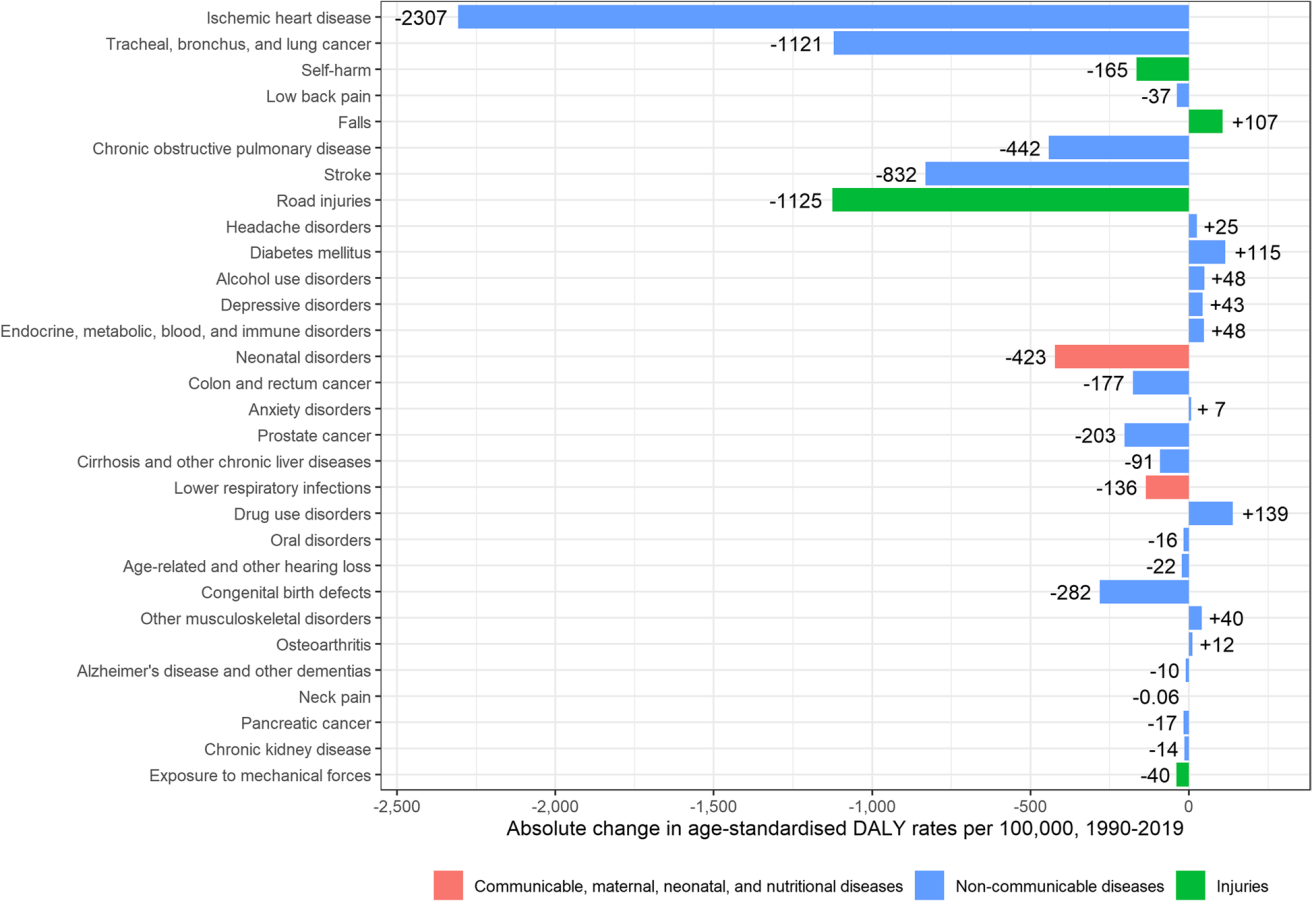
Absolute change in age-standardised Disability-Adjusted Life Year (DALY) rates per 100,000 in men, 1990–2019, Belgium

Age-standardised DALY rates for men significantly decreased from 30,219 DALYs [95% UI: 27,638–33,092] per 100,000 in 1990, to 21,427 DALYs [95% UI: 18,812–24,409] per 100,000 in 2019 (-8,792 DALYs per 100,000). The main contributors to the significant DALY decrease in men were ischaemic heart disease (-2,307 DALYs per 100,000), road injuries (-1,125 DALYs per 100,000), lung cancer (-1,121 DALYs per 100,000), stroke (-832 DALYs per 100,000) and COPD (-442 DALYs per 100,000). On the other hand, DALYs associated with drug use disorders (+ 139 DALYs per 100,000), diabetes mellitus (+ 115 DALYs per 100,000), falls (+ 107 DALYs per 100,000), alcohol use disorders (+ 48 DALYs per 100,000), and endocrine, metabolic, blood, and immune disorders (+ 48 DALYs per 100,000) increased between 1990 and 2019, although the corresponding UIs were overlapping (Fig. 9).

**Fig. 10:**
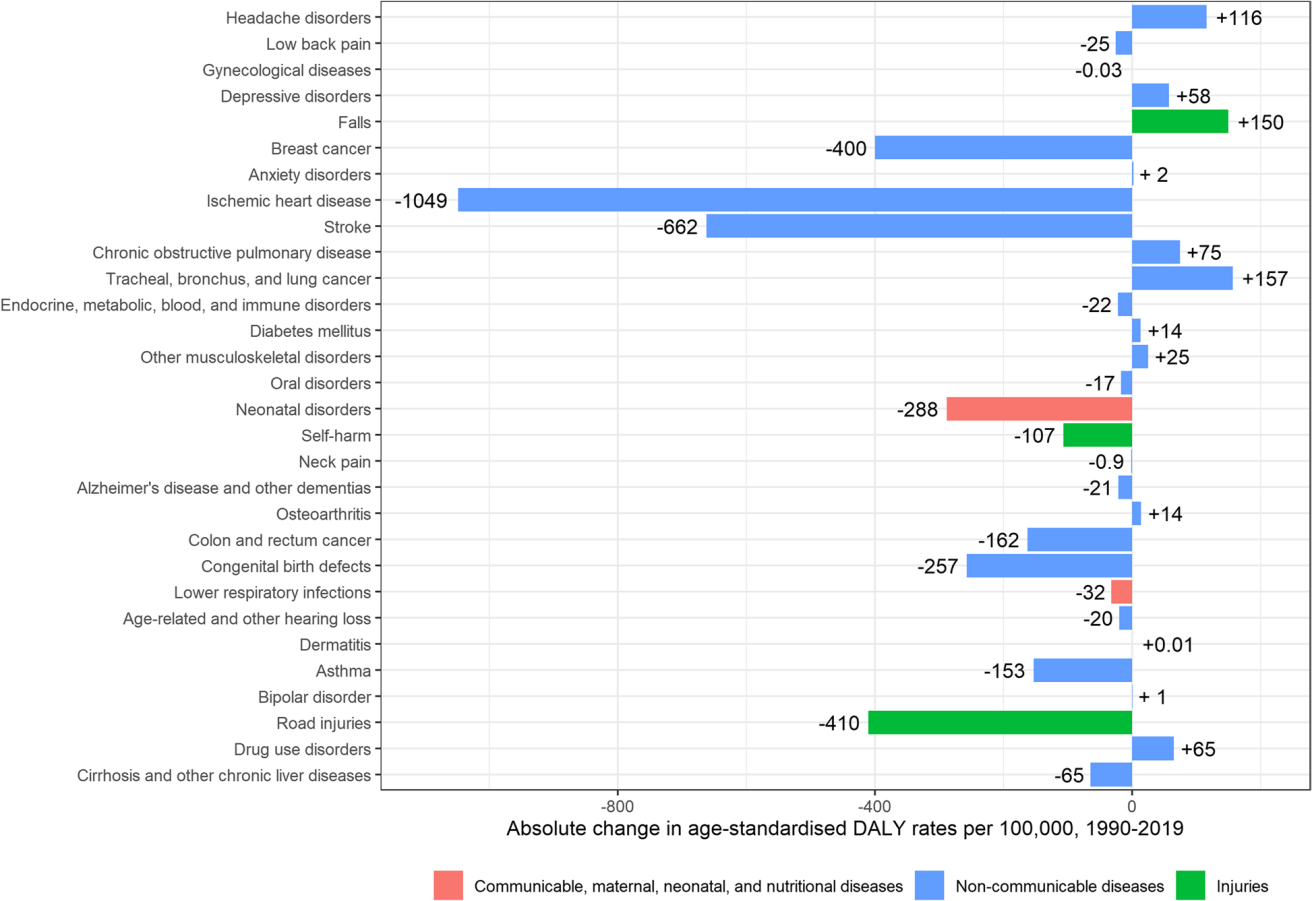
Absolute change in age-standardised Disability-Adjusted Life Year (DALY) rates per 100,000 in women, 1990–2019, Belgium

Age-standardised DALY rates for women also decreased, albeit with overlapping UIs, from 23,203 [95% UI: 20,051–26,847] per 100,000 in 1990, to 19,140 [95%UI: 15,835–22,728] per 100,000 in 2019 (-4,063 DALYs per 100,000; -18%). The main causes of the decrease in DALYs in women were ischaemic heart disease (-1,049 DALYs per 100,000), stroke (-662 DALYs per 100,000), road injuries (-410 DALYs per 100,000), breast cancer (-400 DALYs per 100,000), and neonatal disorders (-288 DALYs per 100,000). DALYs associated with lung cancer (+ 157 DALYs per 100,000), falls (+ 150 DALYs per 100,000), headache disorders (+ 116 DALYs per 100,000), COPD (+ 75 DALYs per 100,000), and drug use disorders (+ 65 DALYs per 100,000), increased between 1990 and 2019 (Fig. 10).

#### Benchmarking

In 2019, the age-standardised DALY rate in Belgium was higher, albeit with overlapping UIs, than the EU-15 average for both men (BE: 21,427 DALYs [95% UI: 18,812–24,409] – EU-15: 20,307 DALYs per 100,000 [95% UI: 17,778–23,174]), and women (BE: 19,140 DALYs [95%UI: 15,835–22,728] – EU-15: 18,521 DALYs per 100,000 [95% UI: 15,328–22,184]). Across the EU-15 countries, Belgium ranked 12^th^ in terms of age-standardised DALY rates for both men and women, respectively. This is a worsening compared to 1990, where Belgium ranked 7^th^ and 8^th^ for men and women, respectively.

In 2019, Belgian men performed significantly worse compared to the EU-15 average in terms of self-harm (+ 365 DALYs per 100,000), lung cancer (+ 262 DALYs per 100,000), COPD (+ 198 DALYs per 100,000). Men however performed significantly better in terms of ischaemic heart disease (-190 DALYs per 100,000). Belgian women performed worse in terms of falls (+ 229 DALYs per 100,000), headache disorders (+ 225 DALYs per 100,000), self-harm (+ 159 DALYs per 100,000), gynaecological disorders (+ 138 DALYs per 100,000), and breast cancer (+ 83 DALYs per 100,000). Women performed better in terms of anxiety and depressive disorders (-122 and -105 DALYs per 100,000, respectively).

Compared to the individual EU-15 countries, Belgian men performed worse than 9 countries in terms of self-harm, and better than 6 countries in terms of ischaemic heart disease. Belgian women performed worse than 6 countries in terms of falls, and better than 6 countries in terms of anxiety disorders.

## Discussion

The health status of Belgian population generally improved between 1990 and 2019. Despite this positive outcome, results show that Belgium did not perform better than other EU-15 countries.

First, LE in Belgium improved between 1990 and 2019 for men and women. The gap between LE for men and LE for women decreased between 1990 and 2019. LE in Belgium was in line with the EU-15 average. This result was supported by the Belgian Health Status report 2019, a report based on national administrative data, registries and surveys. The Belgian Health Status data highlight that the gap between male and female LE has been decreasing over time, reaching the 4.4 years gap observed in 2019 [5]. This decrease in age difference between men and women could be partially explained, in our results, by the drop in ischaemic heart disease and lung cancer-related mortality in men and the significant increase in lung cancer mortality in women.

Second, age-standardised mortality rates in Belgium significantly decreased between 1990 and 2019 for men and women. Despite this improvement, Belgian mortality rates are still above the EU-15 average for both men and women. Mortality caused by ischaemic heart disease decreased between 1990 and 2019; nevertheless, it still accounts for the main cause of death in men and women. Compared to EU-15 average, Belgium performed significantly better in ischaemic heart disease and hypertensive heart disease in both men and women. Nichols and colleagues reported that despite the decrease in coronary heart disease mortality in European countries over the past years, it is still one of the leading causes of death responsible for one in five of all deaths in Europe [20]. In Belgium, therapeutic achievements led to improved outcomes in cardiovascular diseases yet targets are still suboptimal, which can be explained by a healthcare system heavily reliant on acute care and medical interventions [21]. In its 2019 report on the performance of the Belgian health system, the Belgian Health Care Knowledge Centre (“KCE”) has observed that Belgium performed worse than the EU-15 in terms of preventable mortality. The report states that preventable mortality in Belgium is 281.4 deaths per 100,000 in men and 152.4 deaths per 100,000 in women, whereas in the EU-15 it is 263.3 per 100,000 in men and 133.4 per 100,000 in women [1]. According to Hermans and colleagues, for cardiovascular risk factors to be adequately controlled, efforts should focus on lifestyle modifications, patients’ compliance to secondary prevention and clinical adherence to European guidelines [21].

Third, in terms of premature mortality, YLLs in Belgium significantly decreased between 1990 and 2019 for men and women. In 2019, the main causes for premature mortality in men were ischaemic heart disease, lung cancer and self-harm. The main causes for premature mortality in women were breast cancer, ischaemic heart disease and lung cancer. The KCE highlighted that lung cancer is associated with poor prognosis because patients are diagnosed at a relatively late stage [22]. The cancer registry in Belgium reports that lung cancer accounts for the main cause of mortality in cancer among men. They also report that, compared to other cancers, breast cancer is the main cause of mortality in women followed by lung cancer [23]. Looking at head and neck cancer, breast cancer and colorectal cancer, Rosskamp and colleagues showed an association between cancer survival and socioeconomic status [24]. Screening programs and follow up through routine invitation for check-up have been associated with a 20% decrease in breast cancer specific mortality [25]. Belgium introduced a national screening program for breast cancer in 2001. Mammographic screening is free of charge every two years and covers women between 50 and 69 years old [26]. According to the KCE, overall coverage, which includes organized and opportunistic screening, is still suboptimal and covered only 62% of the target population with differences between the three regions. Moreover, socio-economic inequalities remain a barrier for participation in screening programs [1].

Furthermore, alcohol use disorders and liver cancer premature mortality increased between 1990 and 2019. The average consumption of alcoholic beverages is high and remained stable since 2008. Overconsumption, defined as 14 glasses/week for women and 21 glasses/week for men, only declined from 7 to 6% between 1997 and 2018 [5]. In Belgium, hospitalisations account for 82% of the health-related alcohol-attributable direct and indirect costs while only 0.1% of the costs was spent on prevention [27]. Large hospitalisation spending can be tackled by better primary care for earlier disease prevention and empowering healthy behaviours [27–29].

Premature mortality between 1990 and 2019 worsened for drug use disorders in men and lung cancer in women. Compared to the EU-15, Belgium performed significantly worse for self-harm in both men and women. The report on the performance of the Belgian healthcare system shows discouraging mental health indicators such as waiting times for a first contact with ambulatory mental health service, inappropriate prescription of antidepressants and poor adherence to major depression guidelines: Belgian rates are higher than EU-15 countries (Belgium: 79 Defined Daily Doses [DDD] per 1,000 population/day vs. EU-15: 70 DDD per 1,000 population/day) [1].

Fourth, over the last two decades, age-standardised YLDs and Belgium’s ranking among the EU-15 countries have not significantly changed between 1990 and 2019. The main drivers for YLDs were low back pain and headache disorders in both men and women in Belgium in 2019. Age-standardised YLDs for 1990 and 2019 had overlapping UIs, for both men and women. According to the national Health Interview Survey conducted in 2018 in Belgium, low back pain is between the top five chronic conditions most reported among adults with a prevalence of 23% and 26% in men and women respectively [4]. YLDs caused by diabetes have increased between 1990 and 2019 in both men and women. Diabetes is associated with microvascular and macrovascular complications and the risk of developing complications is associated with the duration and severity of diabetes [30]. Thus, diabetic patients need continuous follow-up and monitoring for better glycaemic control to prevent and reduce additional disability. The diabetes type 2 care trajectory in Belgium has resulted in better care quality and follow-up. However, some parameters – such as educators’ referrals – are still underused and should be better exploited. The care trajectory still targets patients with advanced disease stages, rather than early-diagnosis patients: focusing on prevention tackling obesity and healthier diet could be more cost-effective. This is even more important considering that diabetes prevalence is estimated to rise in the next years [31].

Fifth, in terms of DALYs, ischaemic heart disease, lung cancer, and self-harm contributed the most to the burden of disease in men, while headache disorders, low back pain and gynaecological disorders are the top causes for DALYs in women. Streel and colleagues reported a one-year-point prevalence of migraine of 26% with higher prevalence in women [32]. We observed an improvement in DALYs associated with ischaemic heart disease and road injuries between 1990 and 2019 in both men and women and a worsening outcome in drug use disorders for both men and women between 1990 and 2019. Our results are supported by the 2020 report of the Federal Public Service of Transports and Mobility. The report outlined that Belgium recorded a 28% decrease in road fatalities per million inhabitants, a result close to the European average [33].

Between 1990 and 2019, overall age-standardised DALY rates in Belgium have decreased by 23%. This drop in DALYs is mainly due to the decreasing trend in YLL rates, whereas YLD rates have remained stable. A further consequence of these diverging trends is that now disability is the main contributing factor to the burden of disease, with overall YLD rates accounting for 55% of overall DALY rates. In the context of demographic and epidemiologic transitions, governments are challenged by growing healthcare services demands and increasing costs to meet the needs of aging population with multiple chronic conditions. Primary care and sustainable healthcare systems should be adapted toward a life-course approach to chronic diseases and disability [34].

In summary, our results show that the main contributors to mortality and morbidity in Belgium include ischaemic heart disease, lung cancer, and self-harm in men; and headaches, low back pain and gynaecological disorders in women. Self-harm (among men) and falls (among women) pose a higher burden in Belgium than in many other EU-15 countries, indicating a realistic potential for improvement. Falls furthermore rank among the top five causes of disease burden in both men and women, and show an increasing tendency over time, highlighting an emerging priority. Multidisciplinary fall prevention strategies are essential for reducing non-fatal injuries and injury deaths [35]. Headache disorders are the major cause of disease burden among women, and show increasing trends and rates that are higher than many other EU-15 countries. Our results furthermore show a worrisome evolution of lung cancer and COPD in women that could be explained by their smoking behaviours [36]. Among men, lung cancer shows a positive evolution, but remains among the top causes of disease burden, with rates that are higher than many other EU-15 countries. Finally, drug use disorders and diabetes show increasing trends in age-standardised DALY rates, which requires further attention. Given that many of the priority diseases identified in this study are associated with avoidable mortality, we recommend policy-makers to prioritise prevention strategies for early detection and creating healthier living and working environments, to reduce exposure to air pollution and discourage detrimental behavioural risk factors such as tobacco use, alcohol consumption, and unhealthy eating habits [37].

This article is subject to the limitations of GBD studies such as limitations associated with the availability of primary data. GBD studies rely on modelling results estimates when primary data is not available [12]. As a matter of fact, most Belgian health information sources have a delay of 2–3 years [38], which means that many of the GBD 2019 estimates for Belgium rely on projections from other years or neighbouring countries. Furthermore, international differences in registration may play a role when estimating the frequency of non-fatal conditions. Relying on estimations to evaluate country progress might result in false assumptions in health trends and assessment based on projections or estimations. Predicted results cannot sense change in policy or circumstances which makes us question transparency and the ethical side of guiding health agendas based on estimations rather than real values [39]. Furthermore, we were unable to correctly measure statistical significance of our comparisons. Uncertainty in the GBD estimates is characterised using 1000 draws from the posterior model, but these 1000 draws are not publicly available. Instead, we relied on a comparison of the 95% UIs, which is a less robust way for concluding on differences. Specifically, non-overlapping intervals imply significant differences, while overlapping intervals do not necessarily imply insignificant results and remain inconclusive [40].

This study gives us information about health status in Belgium on a countrywide level and compares it to other EU-15 which can help understand health priorities to be addressed on a national level. Nevertheless, many health outcomes revealed to be different between regions and socio-economic classes which may be hindered by country averages [41]. Current GBD estimates however do not provide subnational estimates for Belgium, nor do they integrate health inequalities at country level [12]. Hence, it is important to also assess health performance on a subnational level given the federal organisation and the autonomous competences of the regions in terms of health programs and account for the socio-economic differences. This is currently being achieved via the Belgian national burden of disease study, which complements the results of the GBD study [42].

## Conclusion

Non-communicable diseases remain the main contributors for health burden in Belgium, with disability accounting for an increasingly larger share of the disease burden. Despite considerable improvements, Belgium’s ranking for DALYs decreased between 1990 and 2019 compared to the EU-15. Self-harm and falls are major causes of disease burden, with DALY rates that are higher than in many other EU-15 countries, indicating a realistic potential for improvement. Lung cancer DALY rates remain worrisome for men, and even show an increasing trend for women. Increasing trends of headache disorders, drug use disorders, and diabetes, require further attention. Primary and secondary prevention are crucial elements for reducing the burden of disability on the healthcare system. GBD 2019 estimates are subject to many limitations such as accuracy and external validity. Therefore, national burden of disease studies remain essential for more accurate health estimates and for guiding local health policy.

## Data Availability

All data were retrieved from the GBD study, via the GBD results tool (http://ghdx.healthdata.org/gbd-results-tool). Detailed R code and outputs are available via https://github.com/brechtdv/GBD-BE.

## Abbreviations

BE: Belgium
COPD: Chronic obstructive pulmonary disease
DALY: Disability-adjusted life year
DW: Disability weight
EU: European Union
GBD: Global burden of disease
KCE: Belgian Health Care Knowledge Centre
LE: Life expectancy
UI: Uncertainty interval
YLD: Year lived with disability
YLL: Year of life lost
